# Melatonin administration as a strategy to mitigate weaning stress in Lacaune lambs

**DOI:** 10.3389/fvets.2026.1824547

**Published:** 2026-05-04

**Authors:** María Moreno-Manrique, Joel Bueso- Ródenas, Carlos Mínguez, Carla Ibáñez, Marta González, Xavier Valldecabres, Arantxa Villagrá, Rodolfo Ungerfeld, Aline Freitas-de-Melo

**Affiliations:** 1 Escuela de Doctorado, Department of Animal Production and Public Health, Faculty of Veterinary Medicine and Experimental Sciences, Catholic University of Valencia San Vicente Mártir, Valencia, Spain; 2 Department of Animal Production and Public Health, Faculty of Veterinary Medicine and Experimental Sciences, Catholic University of Valencia San Vicente Mártir, Valencia, Spain; 3 Center for Animal Research and Technology (CITA-IVIA), Valencian Institute for Agricultural Research (IVIA), Segorbe, Castellón, Spain; 4 Departamento de Biociencias Veterinarias, Facultad de Veterinaria, Universidad de la República, Montevideo, Uruguay

**Keywords:** animal behavior, animal stress, dairy sheep, milk replacer, weaning

## Abstract

**Introduction:**

Weaning from artificial milk feeding represents a critical transition in intensively reared dairy lambs, involving dietary change, relocation, and social reorganization, all of which may compromise welfare, immune status, and growth performance. Melatonin, owing to its anxiolytic, antioxidant, and immuno-modulatory properties, has been proposed as a potential strategy to mitigate stress-related responses during this period.

**Methods:**

Sixty Lacaune lambs were randomly assigned at weaning to one of three treatments: control (saline injection), intravenous melatonin (18 mg), or a slow-release subcutaneous melatonin implant. Behavioral patterns were recorded from Day −3 to Day 3 relative to weaning, and body weight was monitored until Day 31. Hematological parameters, serum cortisol, acute phase proteins (CRP and haptoglobin), and fecal *Escherichia coli* counts were assessed on Days −4 and 4.

**Results:**

Subcutaneous melatonin implants attenuated several behavioral indicators of weaning stress, including walking, bleating, and suckling attempts, and were associated with improved post-weaning growth performance from Day 11 onward. Intravenous administration produced limited effects. Cortisol concentrations increased in melatonin-treated lambs on Day 4, although this was not accompanied by adverse behavioral or productive outcomes. Most hematological parameters were unaffected by treatment; however, hemoglobin concentration increased selectively in the implant group. Acute phase proteins and fecal *E. coli* counts did not differ among treatments.

**Discussion:**

Sustained melatonin administration via subcutaneous implants improved behavioral adaptation and post-weaning growth without evidence of detrimental health effects. These findings suggest that slow-release melatonin may represent a promising supportive strategy during the weaning transition in intensive dairy sheep systems.

## 1 Introduction

In intensive dairy sheep production systems, lambs are commonly separated from their mothers shortly after birth to maximize the amount of milk available for dairy processing (1). Consequently, newborn lambs are typically reared on milk replacers delivered through automatic feeding systems (2). However, successful adaptation to these systems requires training (3). Difficulties in learning to use the feeder may lead to digestive disorders and, in severe cases, starvation and death (4). Artificial rearing also impairs immune system development (5) and rumen function (6), compromising the transition to solid feed and reducing growth performance during the post-weaning fattening period (7). Moreover, in large-scale dairy sheep farms, weaning from artificial feeding is often sudden and typically involves transporting lambs to new fattening facilities, where they are housed with unfamiliar lambs of similar body weight (7, 8). The combined effects of suckling cessation, transport, social mixing, sudden dietary transition, and environmental change constitute a highly stressful event for lambs (9). Accordingly, the loss of artificial suckling, associated with a physical relocation, reduces body weight gain and markedly compromises lamb welfare (10), highlighting the need to develop strategies to mitigate these negative consequences. The assessment of these welfare consequences relies on validated behavioral indicators: increased walking, bleating, and suckling attempts have been established as reliable markers of separation distress and weaning-induced agitation in lambs, while a return to baseline activity levels alongside stable or increased eating and ruminating reflects successful behavioral adaptation (9, 10).

To mitigate the impact of weaning-related stress, various strategies have been explored, mainly based on modifying weaning procedures and management practices (9, 11, 12). Pharmacological approaches, however, may also provide effective alternatives. For example, administration of injectable progesterone reduces behavioral indicators of stress in weaned ewes (13), likely through the action of its neuroactive metabolites (14, 15). Another promising alternative is melatonin, a hormone with anxiolytic, antioxidant, anti-inflammatory, and immunomodulatory properties (16). In small ruminants, melatonin is commonly administered as subcutaneous implants to induce cyclic activity during the non-breeding season. Achieving this effect on the female reproductive system requires several weeks of sustained elevated blood melatonin concentrations (17, 18). Besides its application in controlling sheep reproduction (19, 20), several studies have used melatonin in lambs for various purposes. The application of melatonin implants for 15 days in fattening lambs reduces their locomotor activity and both rectal and surface body temperatures (21). The insertion of melatonin implants in 2-month-old male lambs for 60 days promotes growth, increase plasma concentrations of growth hormone and testosterone, and modulates the composition of the intestinal microbiota (22). However, the use of subcutaneous melatonin implants to modulate short-term responses, such as those occurring immediately after weaning, presents certain limitations. To the best of our knowledge, no studies have characterized the rapid changes in circulating melatonin concentrations following implant insertion, as most research has focused on long-term endocrine or reproductive outcomes. Furthermore, the high cost of implants may reduce their practicality when the expected benefit is short-term, such as lowering acute stress responses to weaning from artificial feeding, rather than enhancing reproductive or productive performance. Therefore, it is important to explore alternative administration routes for melatonin, including injectable formulations, which may offer a more efficient and cost-effective approach to addressing acute stress in lambs. In fact, intravenous infusion of melatonin in ewes mitigates both the endocrine and behavioral responses to social isolation, supporting its role as a calming modulator under an acute stressful situation (23). The potential of melatonin to attenuate stress-related responses may be explained by its action at multiple regulatory levels, including anxiolytic effects, modulation of HPA axis activity, and antioxidant and immuno-modulatory properties (16). These mechanisms may collectively contribute to dampening both the behavioral and endocrine responses to acute stressors, as previously demonstrated in sheep under social isolation (23).

Therefore, we hypothesized that administering melatonin to lambs shortly before weaning from artificial feeding attenuates the adverse welfare responses associated with this process. The objective of this study, therefore, was to evaluate whether melatonin, administered either as an intravenous injection or as a slow-release implant, mitigates the effects of weaning from artificial feeding on behavioral indicators, hematological responses, cortisol and acute-phase proteins concentrations, growth performance, and intestinal bacterial load.

## 2 Materials and methods

All experimental procedures were approved by the Animal Experimentation Ethics Committee of the Universidad Católica de Valencia “San Vicente Mártir” (reference code CEEAUCV2012).

### 2.1 Study site and farm management

The study was conducted at a commercial dairy sheep farm in Valencia, Spain (39°16′46″N, 0°34′21″W), in March 2023. This farm managed approximately 5,000 Lacaune ewes, selected for dual-purpose production, milk yield, and lamb fattening. The facilities included mechanical milking systems, designated units for artificial lamb rearing, fattening pens, and a storage area for raw feed materials. Adult sheep were housed in free-stall barns, divided into six areas according to their physiological and productive stages.

During the natural breeding season (September–December, autumn in the Northern hemisphere), the estrous cycle of ewes was synchronized by using intravaginal sponges containing a progesterone analog (17.9 mg of flugestone, Chronogest, MSD Animal Health Spain, Carbajosa de la Sagrada, Spain) that were inserted for 14 days. After sponge withdrawal, ewes were joined with fertile rams fitted with a marking harness to identify mated females, and mating was controlled daily. Lambing was monitored daily during the estimated parturition period. At birth, lambs were immediately separated from the ewes, identified, and treated with umbilical disinfection. During the first 2 days of life, newborn lambs were fed colostrum from the farm’s own reserves, which was previously collected, frozen, and thawed before use. Thereafter, lambs entered the artificial rearing phase, housed in straw-bedded pens measuring approximately 15 m^2^ and grouped in batches of around 60 lambs. They received ad libitum milk replacer (ELVOR 63; 24% crude protein, 24% fat, 5% fiber, 7% ash, 0.9% calcium, 0.45% sodium, 0.75% phosphorus) provided through an automated feeding system (JR, El Torno, Ciudad Real, Spain). In addition to the milk replacer, during this entire period, lambs were provided with straw and starter feed (Lactoiniciacor, Nanta, Silla, Valencia; 18% crude protein, 4% fiber, 3% fat, 6.9% ash, 43% starch and sugars) offered *ad libitum*, along with continuous access to fresh water.

Lambs were weaned from milk substitute at around 25 days of age, as commonly practiced in intensive dairy Lacaune breed lambs in Spain, and moved from the artificial rearing units to fattening pens located 200 m away. The fattening pens had a capacity of approximately 100 lambs and were equipped with individual feeders and drinkers for solid feed and water, respectively. No milk replacer was provided in the fattening pens. The fattening diet consisted of the same starter feed used during artificial rearing (Lactoiniciacor), offered ad libitum.

### 2.2 Animals and experimental design

A total of 60 lambs from the same lambing flock were used in this study. All animals were managed under the farm’s standard husbandry conditions, as previously described. The study included 34 males and 26 females, and excluded lambs with any signs of disease or injury prior to the start of the experiment.

**Figure 1:**
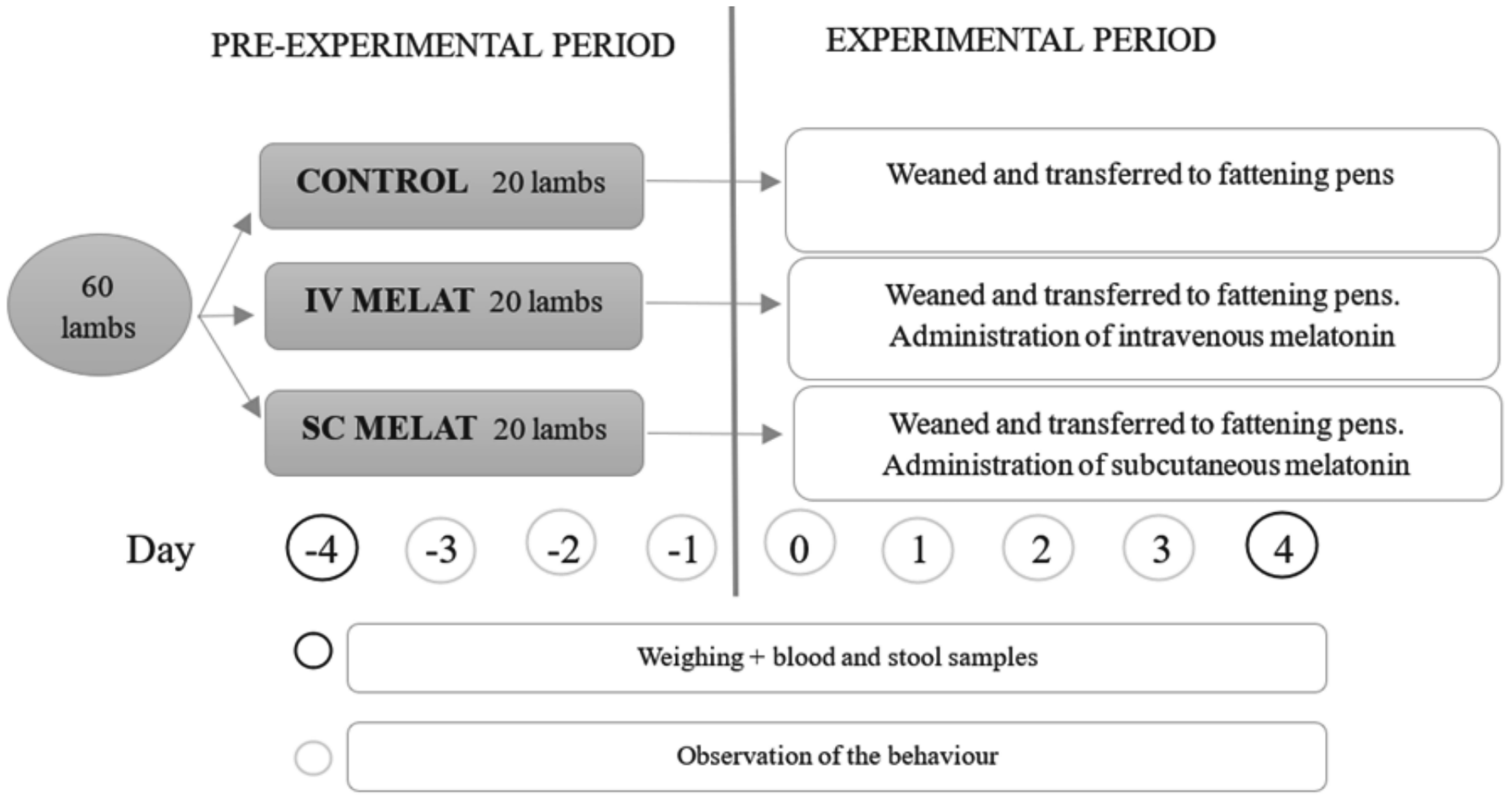
Experimental design. Day 0 corresponds to the day of treatment administration in the diagram. *Weighing controls were also done on days −4, 0, 4, 11, 18, 25, and 31 of the experiment.

The experiment began with a 4-day pre-experimental period during which all lambs were maintained under identical conditions (Figure 1). During this period, lambs were randomly assigned to one of three treatment groups of 20 animals, balanced by body weight and sex, and housed in separate pens by group. Group composition remained stable throughout the entire study. The experimental treatments were applied immediately before weaning (Day 0) and consisted of:

1. Control group (CON group): the lambs received a subcutaneous injection of 2 mL sterile saline solution at the base of the ear.
2. Intravenous melatonin group (IVM group): the lambs received an intravenous bolus of melatonin (18 mg of melatonin dissolved in sterile saline solution for intravenous administration; Sigma-Aldrich, St. Louis, MO, United States), administered at the jugular vein.
3. Subcutaneous melatonin implant group (SCM group): the lambs were treated with an 18 mg slow-release subcutaneous melatonin implant (Melovine, CEVA Santé Animale, Libourne, France), which was inserted at the base of the ear using the applicator provided by the manufacturer.

All treatments were administered in a single session on Day 0. Following the application of the treatments, lambs from all three groups were relocated to the fattening facilities described above and remained there for the duration of the experimental period (Days 0–31).

### 2.3 Lambs’ body weight

Body weight measurements were recorded using a portable digital dynamometer (model SBS-KW-300AG, Steinberg, Berlin, Germany), which allowed for easy transport between pens. Each lamb was weighed individually at the pre-experimental period (Days −4 and 0) and during the experimental period (Days 4, 11, 18, 25, and 31).

### 2.4 Behavioral recordings

Behavioral observations were conducted over a 7-day period, from Day −3 to Day 3, using 15-min scan sampling. Each treatment group (*n* = 20 animals per group) was observed simultaneously by two trained observers stationed outside the pen at a sufficient distance to avoid any influence on lamb behavior. Lambs were individually identified by large numbered marks (1–20) applied with animal-safe marking spray on both flanks, allowing unambiguous identification from a distance. Observations were divided into two daily periods: morning (08:30–11:30) and afternoon (15:30–18:30), avoiding central hours during which farm staff performed routine tasks such as cleaning feeders or adding bedding. Data were recorded every 15 min during a 1-min observation window, yielding 13 observations per period per animal. Over the entire 7-day observation period, a total of 182 scan sampling observations were therefore collected per individual animal (13 observations × 2 periods × 7 days).

Six behaviors were recorded: (i) standing: animal motionless with all four limbs in contact with the ground; (ii) walking: animal actively moving around the pen; (iii) eating: animal consuming solid feed at the feeder or from the floor; (iv) ruminating: animal performing rumination movements; (v) bleating: animal emitting vocalizations; (vi) suckling: animal attempting to suckle from a penmate or a fixture. These behaviors were selected based on their established use as indicators of stress and welfare during the weaning transition in small ruminants (9, 10).

### 2.5 Blood collection

Blood samples were collected from the jugular vein on Days −4 and 4. For each animal, two types of blood samples were obtained using a vacuum tube system: one containing lithium heparin (BD Vacutainer®, Becton, Dickinson and Company, Franklin Lakes, NJ, United States) for hematological analysis, and one without anticoagulant (BD Vacutainer® SST™ II Advance) for serum biochemistry. Samples were immediately placed on ice and transported to the laboratory within 2 h of collection.

### 2.6 Hematological measurements

The blood sample with anticoagulant was used to assess hematological variables, including white blood cell count (WBC), red blood cell count (RBC), hemoglobin concentration (HGB), hematocrit (HCT), mean corpuscular volume (MCV), mean corpuscular hemoglobin (MCH), mean corpuscular hemoglobin concentration (MCHC), corpuscular hemoglobin concentration mean (CHCM), corpuscular hemoglobin (CH), red cell distribution width (RDW), hemoglobin distribution width (HDW), neutrophil count (NEUT), lymphocyte count (LYMPH), monocyte count (MONO), eosinophil count (EOS), and basophil count (BASO). Samples were analyzed using an automated hematology analyzer (ADVIA 120, Siemens Healthineers, Erlangen, Germany), validated for use in sheep (24, 25).

### 2.7 Cortisol and acute-phase protein concentrations

The serum obtained from the second blood sample was used to quantify cortisol, C-reactive protein (CRP), and haptoglobin (HAPTO). Cortisol concentration was measured via competitive ELISA (Salivary Cortisol ELISA, Salimetrics LLC, Carlsbad, CA, United States) This kit has been previously validated for serum cortisol measurement in sheep and dairy cattle (26, 27). CRP and haptoglobin were determined by ELISA following the procedures described by Tothova et al. (28).

### 2.8 Count of *E. coli* colonies from lamb feces

On the same sampling days (Days −4 and 4), fresh fecal samples were manually collected directly from the rectum of each lamb for microbiological analysis. The samples were first plated on MacConkey Agar (bioMérieux, Marcy-l’Étoile, France), according to the procedures described by Collins and Lyne (29), and incubated at 37 °C for 24 h. After incubation, typical *E. coli* colonies were counted and recorded. The count of *E. coli* colonies was used as an indicator of intestinal bacterial load, as previously described for stress-related studies in ruminants (16, 30).

### 2.9 Statistical analysis

Data normality was tested using the Shapiro–Wilk test. To normalize the distribution of *E. coli* counts, data were log10-transformed prior to analysis.

The influence of treatments (CON, IVM, and SCM) on behavioral variables was evaluated using a linear mixed model (PROC GLIMMIX, SAS version 9.2, 2012). The model included treatment as a fixed factor and animal nested within treatment as a random factor. The sampling day and the experimental period (pre-experimental vs. experimental) were also included as fixed factors, along with their interaction with treatment.

For hematological, serum biochemical, and fecal variables, another mixed model (PROC GLIMMIX, SAS 9.2, 2012) was applied. Fixed effects incorporated the treatment group, sampling day (−4 and 4), and their interaction. Animal nested within treatment was included as a random effect.

In all models, time was considered as a repeated measure, and the compound symmetry covariance structure was used to model within-subject correlations over time. Sex was tested in the initial model but excluded from the final analyses due to the absence of significant main effects or interactions.

In summary, behavioral data consisted of scan sampling observations recorded across 7 days (Days −3 to 3), yielding a total of 182 observations per individual animal (13 observations × 2 daily periods × 7 days), with 20 animals per treatment group. Hematological, serum biochemical, and fecal variables were assessed at two time points (Days −4 and 4), with one observation per animal per sampling day (*n* = 20 per group per time point). Body weight was recorded at 7 time points (Days −4, 0, 4, 11, 18, 25, and 31), with one measurement per animal per weighing day (*n* = 20 per group).

## 3 Results

### 3.1 Body weight

**Figure 2:**
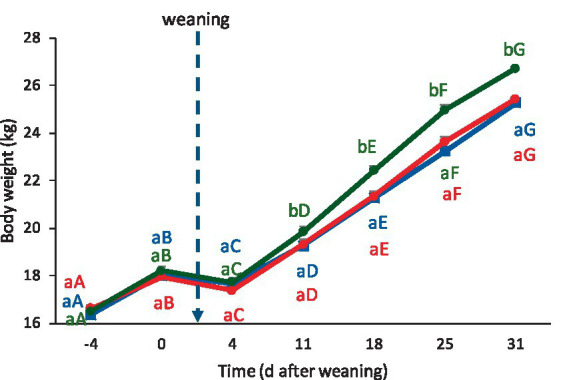
Evolution of body weight (mean ± SE) in lambs from Day −4 to Day 31 relative to weaning. Blue line: CON (control group); Red line: IVM (intravenous melatonin group); Green line: SCM (subcutaneous melatonin implant group). Different lowercase letters indicate significant differences among treatments within the same day (*p* < 0.05). Different uppercase letters indicate significant differences across days within the same treatment (*p* < 0.05).

There was a significant interaction between treatments and time in the progression of lambs’ body weight (*p* = 0.013). Before weaning from milk substitute (Days −4 and 0), there were no differences in body weight among groups (*p* = 0.37). There was a slight decrease in body weight from Day 0 to Day 4 in all groups, followed by significant increases on each subsequent recording day (*p* ≤ 0.05). However, from Day 11 onwards, SCM lambs were heavier than CON and IVM lambs in all subsequent recordings (*p* ≤ 0.05) (Figure 2).

### 3.2 Behavioral recordings

**Figure 3:**
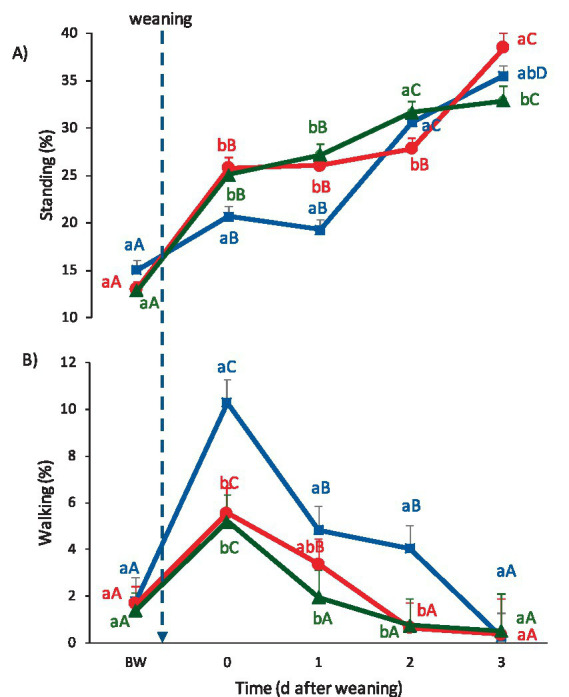
Behavioral responses to weaning (mean ± SE) from Day −3 to Day 3 relative to weaning: **(A)** Standing and **(B)** walking. Blue line: CON (control group); Red line: IVM (intravenous melatonin group); Green line: SCM (subcutaneous melatonin implant group). Different lowercase letters indicate significant differences among treatments within the same day (*p* < 0.05). Different uppercase letters indicate significant differences across days within the same treatment (*p* < 0.05).

**Figure 4:**
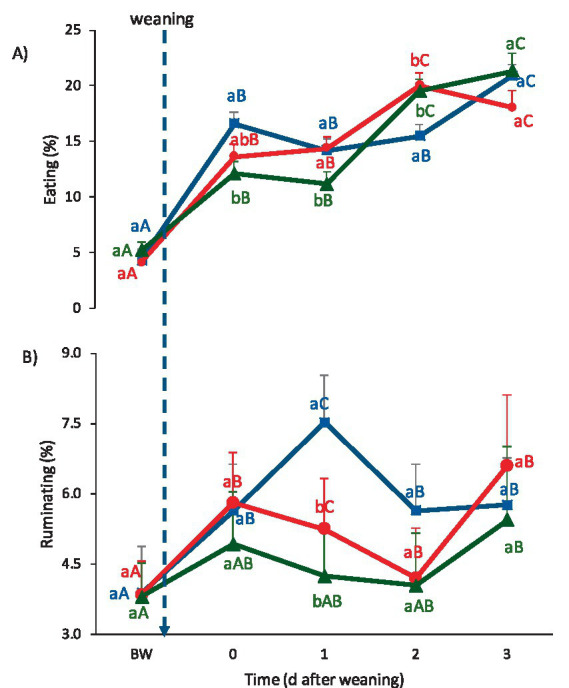
Feeding-related behaviors (mean ± SE) from Day −3 to Day 3 relative to weaning: **(A)** Eating and **(B)** ruminating. Blue line: CON (control group); Red line: IVM (intravenous melatonin group); Green line: SCM (subcutaneous melatonin implant group). Different lowercase letters indicate significant differences among treatments within the same day (*p* < 0.05). Different uppercase letters indicate significant differences across days within the same treatment (*p* < 0.05).

There were significant interactions between treatments and time in all the recorded behaviors (standing, walking, eating, and suckling: *p* ≤ 0.0001 for all; ruminating: *p* = 0.016; bleating: *p* = 0.0008. There were no significant differences in any behavior before weaning from the milk substitute (Figures 2–4).

The proportion of observations in which lambs were standing up was greater on Days 0, 1, and 2 in CON and SCM lambs than in IVM lambs (*p* ≤ 0.05), with no differences among treatments on Day 3 (Figure 3A).

The proportion of time that lambs walked increased widely on Day 0 in all the groups (*p* ≤ 0.05), but more sharply in CON lambs, achieving greater values than both melatonin-treated groups (Figure 3B). There were no differences between IVM and SCM lambs on Day 0. On Day 1, SCM lambs reduced time spent walking, returning to baseline values. On the other hand, although IVM and CON lambs also reduced walking time, they did not return to the initial values. On Day 1, CON lambs still walked more frequently than SCM lambs (*p* < 0.05), while IVM lambs showed intermediate values, which did not differ significantly from those of the other two groups. On Day 2, CON lambs still walked more than both melatonin-treated groups (*p* ≤ 0.05), with no differences between the two treated groups. At this point (Day 2), while both melatonin-treated groups walked similarly to before weaning at this time (Day 2), CON lambs reached similar values only on Day 3.

The proportion of observations in which lambs ate and ruminated is presented in Figures 4A,B, respectively. The three treatments increased time spent eating on Day 0, although the values were greater in CON and IVM lambs than in SCM lambs (*p* ≤ 0.05). On Day 1, eating frequency remained stable, and on Day 2, it increased again relative to Day 1 in both melatonin-treated groups (*p* ≤ 0.05), achieving greater values than those of CON lambs, with no differences between IVM and SCM lambs. On Day 3, the three groups displayed similar eating frequencies.

The frequency of ruminating also increased on Day 0 across all groups. On Day 1, the frequency increased sharply in CON lambs, achieving values greater than those of SCM and IVM lambs (*p* ≤ 0.05), with no difference between the two melatonin-treated groups. On Day 2, CON lambs reduced the time spent ruminating to levels comparable to those of the melatonin-treated groups, and remained stable through Day 3.

**Figure 5:**
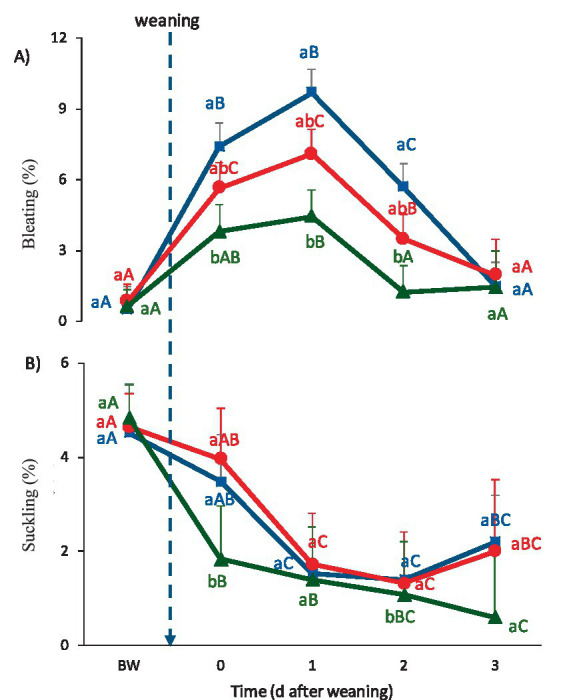
Stress-related behaviors (mean ± SE) from Day −3 to Day 3 relative to weaning: **(A)** Bleating and **(B)** Suckling attempts. Blue line: CON (control group); Red line: IVM (intravenous melatonin group); Green line: SCM (subcutaneous melatonin implant group). Different lowercase letters indicate significant differences among treatments within the same day (*p* < 0.05). Different uppercase letters indicate significant differences across days within the same treatment (*p* < 0.05).

Bleating increased in CON and IVM lambs on Day 0 (*p* ≤ 0.05), and remained elevated on Day 1. However, SCM lambs showed an increase only on Day 1 compared with the pre-weaning period (*p* ≤ 0.05) (Figure 5A). Although CON lambs diminished the bleating frequency on Day 2 (*p* ≤ 0.05), they still bleated more than SCM lambs, while IVM lambs showed an intermediate frequency that did not differ from either CON or SCM lambs. On Day 2, all treatments returned to pre-weaning values.

The frequency of suckling attempts decreased in all the groups on Day 0, but the reduction was greater in IVM lambs than in SCM and CON lambs (*p* ≤ 0.05), resulting in lower values on Day 0 (Figure 5B). On Day 1, both CON and IVM lambs reduced the frequency of suckling attempts (*p* ≤ 0.05), thereby eliminating treatment differences. However, on Days 2 and 3, CON and IVM lambs again displayed a greater frequency of suckling attempts than SCM lambs (*p* ≤ 0.05).

### 3.3 Cortisol, C-reactive protein, haptoglobin, and *E. coli*

A significant interaction between treatment and time was observed in cortisol concentration (*p* = 0.004). While concentrations did not differ significantly among groups before weaning from milk substitute, on Day 4, cortisol concentration was greater in both melatonin-treated groups than in CON lambs (*p* ≤ 0.05), with no differences between melatonin-treated groups. Interestingly, cortisol concentration increased in both melatonin-treated groups on Day 4 (*p* ≤ 0.05), whereas no changes were observed in the CON lambs. There was also an interaction between treatment and time in CRP concentration (*p* ≤ 0.05). Before weaning from the milk substitute, CRP concentration was greater in CON than in IVM lambs. In both melatonin-treated groups, CRP concentration increased after weaning, so at that moment, CRP concentrations did not differ among treatments. There was a pronounced increase in haptoglobin concentrations after weaning from milk substitute (*p* ≤ 0.05), with no differences among treatments. The treatments, time, and their interaction did not affect *E. coli* count (Table 1).

**Table 1:** Mean ± standard error (SE) of biochemical parameters, and E coli counts in feces, in the three groups of lambs studied, pre- and post-treatment.

Parameter	Pre-experimental values			Experimental values		
	Control	IV melat	SC melat	Control	IV melat	SC melat
CORT (ng/mL)	2.43 ± 0.58 aA	3.27 ± 1.68 aA	2.26 ± 0.42 aA	2.75 ± 0.56 aA	8.83 ± 1.9 bB	6.16 ± 1.0 bB
CRP (pg/mL)	47.20 ± 2.52 aA	40.19 ± 1.56 bA	43.14 ± 2.47 abA	54.13 ± 2.28 bA	52.56 ± 2.66 bB	51.64 ± 3.97 bB
HAPTO (mg/mL)	0.38 ± 0.08 aA	0.38 ± 0.05 aA	0.34 ± 0.07 aA	0.64 ± 0.10 aB	0.55 ± 0.10 aB	0.63 ± 0.13 aB
Log *E. coli* (Ufc/g)	7.58 ± 0.65 aA	7.44 ± 0.62 aA	8.31 ± 0.63 aA	7.87 ± 0.61 aA	7.23 ± 0.62 aA	7.52 ± 0.65 aA

CORT, cortisol; CRP, C-reactive protein; HAPTO, haptoglobin.

### 3.4 Hematology: red blood cells, hematocrit, and hemoglobin

Red blood cell count and HCT increased on Day 4, and MCV decreased (*p* ≤ 0.05 for all), with no differences among treatments or interaction between treatments and time. Red cell distribution width was not affected by treatments, time, or their interaction. There was an interaction between treatments and time in hemoglobin concentration (*p* ≤ 0.05). While it increased after weaning from milk substitute in SCM, it did not change in CON or IVM lambs (*p* ≤ 0.05) (Figure 5A). Mean corpuscular hemoglobin decreased, and CHCM increased on Day 4 (*p* ≤ 0.05 for both), without effects of treatments or their interaction with time. Mean corpuscular hemoglobin concentration, CH, and HDW did not change with treatments, time, or their interaction (Table 2).

**Table 2:** Mean ± standard error (SE) of hematological parameters, in the three groups of lambs studied, pre- and post-treatment.

Parameter	Pre-experimental values			Experimental values		
	Control	IV melat	SC melat	Control	IV melat	SC melat
RBCC (10^6^ cells/μL)	9.73 ± 0.36 aA	10.40 ± 0.34 aA	10.11 ± 0.34 aA	11.51 ± 0.33 aB	12.02 ± 0.34 bB	12.25 ± 0.36 bB
HCT (%)	30.11 ± 0.98 aA	31.47 ± 0.98 aA	29.20 ± 1.03 aA	32.79 ± 0.96 aB	34.01 ± 0.98 aB	34.26 ± 1.04 aB
MCV (fL)	30.39 ± 0.73 aA	30.65 ± 0.71 aA	29.89 ± 0.72 aA	28.70 ± 0.70 aB	28.61 ± 0.71 aB	28.00 ± 0.74 aB
RDW (%)	20.72 ± 0.50 aA	21.01 ± 0.48 aA	20.63 ± 0.49 aA	20.15 ± 0.48 aA	21.06 ± 0.48 aA	20.50 ± 0.51 aA
HGB (g/dL)	9.68 ± 0.32 aA	9.98 ± 0.30 aA	9.69 ± 0.30 aA	9.98 ± 0.30 aA	10.45 ± 0.30 aA	10.44 ± 0.32 aB
MCH (pg)	9.98 ± 0.33 aA	9.70 ± 0.32 aA	9.64 ± 0.32 aA	8.77 ± 0.31 aB	8.74 ± 0.32 aB	8.57 ± 0.33 aB
CHCM g/dL	30.63 ± 0.19 aA	30.73 ± 0.19 aA	30.97 ± 0.19 aA	31.52 ± 0.19 aB	31.43 ± 0.19 aB	31.59 ± 0.20 aB
MCHC (g/dL)	29.93 ± 0.76 aA	31.45 ± 0.72 aA	32.24 ± 0.72 aA	30.44 ± 0.70 aA	30.30 ± 0.72 aA	30.51 ± 0.76 aA
CH (pg)	9.28 ± 0.22 aA	9.38 ± 0.22 aA	9.23 ± 0.22 aA	9.03 ± 0.22 aA	9.01 ± 0.22 aA	9.04 ± 0.23 aA
HDW (g/dL)	2.19 ± 0.05 aA	2.09 ± 0.05 aA	2.14 ± 0.05 aA	2.21 ± 0.04 aA	2.17 ± 0.05 aA	2.13 ± 0.05 aA
WBCC (10^3^ cells/μL)	7.75 ± 0.73 aA	6.98 ± 0.69 aA	6.22 ± 0.70 aA	6.64 ± 0.68 aA	8.05 ± 0.69 aA	7.43 ± 0.73 aA
NEUT (10^3^ cells/μL)	2.45 ± 0.51 aA	2.71 ± 0.49 aA	2.25 ± 0.49 aA	2.24 ± 0.48 aA	3.35 ± 0.49 aA	2.55 ± 0.52 aA
LYMPH (10^3^ cells/μL)	3.31 ± 0.35 aA	3.34 ± 0.33 aA	3.24 ± 0.33 aA	3.24 ± 0.33 aA	3.61 ± 0.33 aA	3.81 ± 0.35 aA
MONO (10^3^ cells/μL)	0.33 ± 0.06 aA	0.31 ± 0.06 aA	0.30 ± 0.06 aA	0.29 ± 0.06 aA	0.28 ± 0.06 aA	0.29 ± 0.06 aA
EOS (10^3^ cells/μL)	0.38 ± 0.06 aA	0.44 ± 0.06 aA	0.38 ± 0.06 aA	0.70 ± 0.05 aB	0.65 ± 0.06 aB	0.63 ± 0.06 aB
BASO (10^3^ cells/μL)	0.09 ± 0.01 aA	0.08 ± 0.01 aA	0.08 ± 0.01 aA	0.08 ± 0.01 aA	0.07 ± 0.01 aA	0.07 ± 0.01 aA

RBCC, red blood cell count; HCT, hematocrit level; MCV, mean corpuscular volume; RDW, red cell distribution width; HGB, hemoglobin; MCH, mean corpuscular hemoglobin; CHCM, corpuscular hemoglobin concentration mean; MCHC, mean corpuscular hemoglobin concentration; CH, corpuscular hemoglobin; HDW, hemoglobin distribution width; WBCC, white blood cell count; NEUT, neutrophils count; LYMPH, lymphocytes count; MONO, monocytes count; EOS, eosinophils count; BASO, basophils count. Different lowercase letters indicate significant differences among treatments within the same day (*p* < 0.05). Different uppercase letters indicate significant differences across days within the same treatment (*p* < 0.05).

### 3.5 Hematology: white blood cells

Total white blood cell, neutrophil, lymphocyte, monocyte, and basophil counts were not affected by treatments, time, or their interaction. Eosinophil count increased on Day 4 (*p* ≤ 0.0001), with no differences among treatments or their interaction with time (Table 2).

## 4 Discussion

Subcutaneous slow-release melatonin implants attenuated key behavioral indicators of weaning stress in artificially reared lambs, specifically walking, bleating, and suckling attempts, and enhanced post-weaning growth performance, indicating improved behavioral and productive adaptation to the transition from milk substitute to solid feeding. Intravenous melatonin produced more limited and transient behavioral effects, without demonstrable benefits on growth performance. Minimizing the adverse consequences of weaning is critical not only from an ethical perspective but also for optimizing health and productive performance. Effective mitigation of weaning-related stress, whether through improved handling practices, environmental enrichment, nutritional adjustments, or pharmacological interventions, is therefore essential to support animals’ ability to adapt without compromising health or productivity (9). As previously described, melatonin acts at multiple regulatory levels in mammals, exerting anxiolytic effects and dampening stress responses (16). In diurnal species such as sheep, melatonin also promotes rest and may prolong periods of reduced activity (21), contributing to behavioral stabilization during stressful transitions such as weaning.

The insertion of subcutaneous melatonin implants providing sustained hormone release had several benefits for artificially reared lambs during the weaning transition. SCM lambs recovered more quickly from weaning-related behavioral disturbances and grew faster than those in the other treatment groups. The use of melatonin implants markedly reduced bleating frequency and time spent walking, both energetically costly behaviors associated with separation distress (31–33). However, since walking and standing were mutually exclusive, the inconsistent treatment differences observed in standing behavior across days, together with the higher walking values recorded in IVM lambs, suggest that these results should be interpreted with caution and could not allow firm conclusions regarding the effect of melatonin on locomotor activity. One of the most relevant findings was that both melatonin-treated groups showed reduced suckling attempts directed at other lambs, with SCM animals displaying the most pronounced decline; the attenuation of these stereotyped behaviors is considered an indicator of improved welfare in young ruminants (34, 35). Eating and ruminating frequencies in SCM lambs require careful interpretation: although lower than in controls, these differences do not necessarily reflect reduced intake or impaired digestive function. It is possible that melatonin might synchronize digestive activity with optimal circadian phases, potentially concentrating feeding effort outside observation windows and thereby enhancing overall efficiency without increasing behavioral frequency (36). This interpretation is consistent with the observation that behavioral changes do not always mirror productive outcomes (37), and is supported by the superior growth performance of SCM lambs discussed below.

On the other hand, intravenous melatonin bolus administration produced some short-term behavioral modulation but did not achieve the sustained welfare and productive benefits associated with slow-release subcutaneous administration. This contrast is likely explained by pharmacokinetic differences: subcutaneous implants maintain supraphysiological plasma concentrations for several weeks (18), whereas a single intravenous bolus produces a rapid transient peak followed by clearance. If the modulatory effects of melatonin on the HPA axis, behavioral reactivity, and metabolic efficiency require sustained exposure rather than a brief hormonal surge, the limited effectiveness of the bolus is mechanistically coherent. Considering that intravenous melatonin does mitigate endocrine and behavioral responses to acute stressors such as social isolation in ewes (23), further studies exploring higher doses or repeated bolus administration are warranted to clarify the dose–response relationship in this context. Overall, our findings indicate that commercial subcutaneous melatonin implants are practical to use and exert clear positive effects on lamb welfare and growth performance during the weaning transition.

The post-weaning period is frequently associated with a transient reduction in growth rate due to dietary shifts, environmental changes, and stress-related metabolic alterations (9, 10). Consistent with this, all lambs in the present study experienced a slight weight loss immediately after weaning from milk substitute. SCM lambs were more than 1 kg heavier than controls by Day 25 and maintained this advantage through the end of the trial. This sustained growth benefit suggests that continuous melatonin availability through implants supports better physiological adaptation to weaning, likely through improved metabolic efficiency, reduced catabolic stress responses, and possible modulation of appetite and digestive function (22, 36). The subtle differences between our findings and those of Viola et al. (21) may in part reflect the different time of year in which the studies were conducted and the consequent differences in endogenous melatonin levels in the animals. In contrast, intravenous administration did not improve growth performance over controls, further supporting the conclusion that sustained hormonal exposure is required for melatonin to exert its anabolic effects.

The behavioral responses observed across all experimental groups were characterized by increased activity and bleating after weaning, confirming lambs’ sensitivity to the combined stressors of dietary change and relocation, as previously reported in similar contexts (10). The pattern of behavioral modifications observed in control lambs was consistent with those reported after weaning from both natural suckling and artificial feeding in previous studies (38–41), highlighting the need to develop specific mitigation strategies tailored to the particularities of dairy sheep systems that rely on artificial rearing.

Contrary to the general pattern of beneficial effects, cortisol concentrations increased more in both melatonin-treated groups than in controls after weaning. Interpretation of this finding is constrained by the sampling design: only a single blood sample was collected 4 days after weaning to avoid interference with behavioral observations. As cortisol reflects acute stress responses but is less informative about their prolonged consequences, this strategy may have missed the transient peaks occurring in the first hours post-weaning, as previously reported in sheep (13). It is therefore not possible to determine whether the acute cortisol response was modified by melatonin or whether the elevated values detected at Day 4 in treated animals reflect a delayed adaptive process that had already returned to baseline in controls. Previous studies have indicated that melatonin may modulate rather than suppress cortisol responses under conditions of complex or sustained stress (23), and elevated cortisol does not necessarily indicate impaired welfare or reduced performance when considered in isolation (42, 43). Importantly, the dissociation between elevated cortisol and improved behavioral and productive outcomes in melatonin-treated lambs illustrates that these markers do not always reflect the same underlying processes, and underscores the need to interpret physiological and behavioral data jointly.

Regarding hematological variables, most of them remained within reference intervals and showed no significant differences between treatment groups, indicating that lambs maintained adequate health status throughout the trial. Hemoglobin concentration increased in SCM lambs after weaning, whereas no changes were observed in CON or IVM animals. This selective increase in hemoglobin may suggest a subtle functional modulation of erythrocyte physiology under sustained melatonin exposure. Previous studies have reported positive effects of melatonin on hematological responses in ruminants (44, 45), and its antioxidant properties could enhance erythrocyte stability during management-related stressors such as handling and relocation (46, 47). Mean corpuscular volume decreased during the experimental period across all groups. Importantly, an identical reduction was previously observed in all groups of a study on the same farm, including the group that was neither weaned nor relocated (10), indicating that this change reflects a physiological maturation process associated with advancing age rather than a specific response to weaning stress, consistent with the progressive age-related decrease in erythrocyte volume documented in lambs (46). MCV, therefore, does not appear to be a reliable marker of weaning-related stress in animals of this age. Eosinophil counts increased in all three experimental groups after weaning. In the previous study conducted on the same farm (10), eosinophil counts increased in both the group weaned without relocation and the group weaned and relocated, but decreased in the group that continued receiving milk replacer without any management change. This pattern identifies dietary transition as the primary driver of eosinophilia, independently of relocation stress, consistent with the intestinal antigenic stimulation associated with the shift from milk replacer to solid feed. This interpretation also resolves the apparent paradox with the elevated cortisol in melatonin-treated animals: the eosinophilia here reflects a dietary rather than a stress-mediated mechanism and therefore does not follow the eosinopenic pattern typically associated with glucocorticoid elevation. Acute-phase proteins CRP and haptoglobin increased post-weaning in all groups with no differences between treatments, consistent with a nonspecific inflammatory response to the management transition. Fecal *E. coli* colony counts remained stable across all groups and sampling points, indicating that intestinal bacterial load was unaffected by weaning or melatonin treatment. It should be noted that *E. coli* counts represent a specific marker of enteric bacterial load rather than a comprehensive health indicator; the full hematological and biochemical panel analyzed in this study collectively provides a broader assessment of health status, none of which revealed evidence of clinical pathology in any group.

## 5 Conclusion

Weaning from artificial milk feeding induced marked behavioral and physiological adjustments in Lacaune lambs, confirming the challenging nature of this management transition. Administration of melatonin through a slow-release subcutaneous implant attenuated several stress-related behaviors and enhanced post-weaning growth performance, suggesting improved adaptive capacity. In contrast, a single intravenous bolus produced limited effects, highlighting the importance of sustained hormone availability. Overall, sustained melatonin administration appears to support behavioral stability and growth during the weaning transition and may represent a promising complementary tool to improve welfare in intensive dairy sheep production systems.

## Data Availability

The raw data supporting the conclusions of this article will be made available by the authors, without undue reservation.
